# FPGA-Based Sensors for Distributed Digital Manufacturing Systems: A State-of-the-Art Review

**DOI:** 10.3390/s24237709

**Published:** 2024-12-02

**Authors:** Laraib Khan, Sriram Praneeth Isanaka, Frank Liou

**Affiliations:** Department of Mechanical and Aerospace Engineering, Missouri University of Science and Technology, Rolla, MO 65409, USA; lktmy@mst.edu (L.K.); sihyd@mst.edu (S.P.I.)

**Keywords:** distributed digital factories, additive manufacturing, traditional manufacturing, FPGA sensor, traditional sensors, sustainability

## Abstract

The combination of distributed digital factories (D^2^Fs) with sustainable practices has been proposed as a revolutionary technique in modern manufacturing. This review paper explores the convergence of D^2^F with innovative sensor technology, concentrating on the role of Field Programmable Gate Arrays (FPGAs) in promoting this paradigm. A D^2^F is defined as an integrated framework where digital twins (DTs), sensors, laser additive manufacturing (laser-AM), and subtractive manufacturing (SM) work in synchronization. Here, DTs serve as a virtual replica of physical machines, allowing accurate monitoring and control of a given manufacturing process. These DTs are supplemented by sensors, providing near-real-time data to assure the effectiveness of the manufacturing processes. FPGAs, identified for their re-programmability, reduced power usage, and enhanced processing compared to traditional processors, are increasingly being used to develop near-real-time monitoring systems within manufacturing networks. This review paper identifies the recent expansions in FPGA-based sensors and their exploration within the D^2^Fs operations. The primary topics incorporate the deployment of eco-efficient data management and near-real-time monitoring, targeted at lowering waste and optimizing resources. The review paper also identifies the future research directions in this field. By incorporating advanced sensors, DTs, laser-AM, and SM processes, this review emphasizes a path toward more sustainable and resilient D^2^Fs operations.

## 1. Introduction to Distributed Digital Factory (D^2^F)

A distributed digital factory (D^2^F) is a collection of globally dispersed digital assets and methods enabling 3D projection and overseen by a near-real-time data management system [1,2]. In layman’s terms, a D^2^F is a virtual representation of a real factory created and depicted using digital technologies such as digital twins (DTs), IoT, Artificial Intelligence (AI) and Machine learning (ML), big data analysis and cyber-security [1,3]. Such systems are incorporated with interoperability, real-time-monitoring, feedback, control and optimization. Traditionally, all of these steps were carried out manually, making the process exceedingly time-consuming and nearly impossible to coordinate if distributed over large geographic locations. Today’s digital technologies though have enabled human beings to process all of this information digitally, making it faster, more precise, and easily available. This transformation permits for the automation of intricate tasks, near-real-time data analysis, and communication across networks to enable the creation of D^2^Fs.

The development of distributed manufacturing, a concept similar to D^2^F, can be understood with the advancements in production management. The foundational research in manufacturing and production systems has centered on the centralized manufacturing model that gained importance in the early 20th century [4]. In the latter half of the 20th century, alternative approaches to traditional production methods gained attention. For instance, in Ref. [5], it is noted that the operating systems proposed for tailored products are intrinsically adaptive. Similarly, the authors in Ref. [6] recognized strategic manufacturing strategy decisions, including considerations such as size, capacity, and location. Later in Refs. [7,8], the authors illustrated production as progressively managed within connected networks implying cross-border relationships tied to ownership, internationalization, and location. With the elevation of new technologies, these traditional frameworks require reevaluation to associate with the distributed manufacturing paradigm. Geographic dispersion is an eminent element of distributed manufacturing, which has been affected by globalization recently. The value chain has been increasingly broken down into smaller components and processes, with manufacturing dispersed across several sites [9,10]. This provincial distribution has transmuted over time to have more profound implications. It has gone beyond the physical distance between firm’s divisions to include production systems that function as linked networks [11,12]. As this notion grew, cooperation among enterprises became the norm, resulting in well-structured supply chain networks. These networks feature particular enterprises cooperating to yield products and services [13]. Such designs enabled SMEs to integrate into a sizeable industrial value chain [14].

The increased demand for customized product variants, along with localized manufacturing, imposes imaginative production approaches that challenge conventional processes [15]. Small, flexible, and scalable distributed manufacturing units are envisioned to satisfy the needs of modern operations, including in-time delivery, flexible alterations to production capability and functionality as per customer demand, and environmentally responsible production and supply chains. Distributed manufacturing differs from typical mass production in terms of both scale and location, as well as the relationship between customers and producers. This trend moves from long supply chains and centralized production to a dispersed system centered on proximity, approachability, and local value creation. The user interface is developing, blurring the line between consumers and producers, also known as “prosumers” [16]. Digitalization and the Internet have permitted people to contribute to product design, resulting in more personalization and customization. Along with technical developments, a culture of shared community manufacturing spaces is establishing, with distributed manufacturing having the capability to modify both industrial and urban settings.

Innovative business models within “distributed economies” are the component of the distributed manufacturing paradigm [17]. By using localized resources, these models will practice small-scale and adaptable networks to support sustainable production practices, including resource and energy-efficient manufacturing systems [10,18,19]. These developments are coherent with prospective supply chain that progressively address resource limitation [19,20]. The circular economy, highlighting resources revival and recycling while reducing the energy and ecological effect of extraction and processing, impacts this change [21]. Distributed manufacturing corresponds to a step toward democratizing and distributing manufacturing, consistent with circular economy models. To achieve the collective and cooperative manufacturing abilities that distributed manufacturing is known for, appropriate standards and procedures must be established. Manufacturing is more than just producing goods; it is a collaborative effort in which multiple individuals contribute in a structured process to generate products using specified and measurable techniques. The difference between traditional and D^2^F environments is the transition from individual worker expertise to the utilization of advanced procedures and digital technologies. These technologies permit various people to join in manufacturing processes across multiple locations in a consistent manner.

In recent years, industries have been transformed by the introduction of cutting-edge technology enabling the industry 4.0 paradigm and ushering in Industry 5.0 [22]. The D^2^F has evolved as an entirely new manufacturing paradigm, capable of fundamentally transforming the manufacturing industry by leveraging advanced manufacturing and digital techniques, including laser-based additive manufacturing (laser-AM), material removal processes (MRPs), advanced sensors, and digital twins (DTs) into one holistic factory management solution of the future. The D^2^F mission is to improve the entire manufacturing process, beginning from design to production, inspection and quality control, by creating a seamlessly connected manufacturing ecosystem.

In D^2^F, existing analog systems from Traditional Factories (TF) have been replaced by the digital systems supporting a wide range of manufacturing technologies and equipment. One of these manufacturing technologies called Laser-AM printing process utilizes a laser beam to melt feedstock and deposit freeform shapes in a layer-by-layer fashion, allowing to manufacture near net shape parts and the repair complex geometries [23]. Hence, it has even been categorized as a crucial manufacturing process in the advancement of D^2^F [24]. Compared to laser-AM process, conventional subtractive processes like MRPs remove material from a workpiece and have been widely adopted in a range of industries [25]. Advancing sensing devices integrated with the manufacturing process as well as DTs, are critical for D^2^F as they provide continuous operational monitoring, and this data can then be utilized for feedback and control [26,27]. Advancing sensing devices can generate real-time data and be used to monitor a manufacturing process using any number of process critical variables such as thermal distribution, humidity, vibrations etc. [28]. Advancing sensing devices are considered a vital enabling technology in D^2^F, helping to improve product quality by monitoring process variables, propose preventative actions in near real-time and thereby lessen maintenance downtime; thus, elevating the reliability of a manufacturing process.

Field-Programmable Gate Arrays (FPGA)-based advanced sensing devices can be considered as essential components in a digital factory due to their unique features, including flexible design and re-design, low power consumption compared to traditional sensors, coupled with high-speed processing of large datasets [29,30]. FPGAs are programmable and can simultaneously manage intricate algorithms as well as signal processing during a manufacturing process; thus, allowing quicker process control and optimization [31]. The FPGA-based Advancing sensing devices can improve the ability to detect process inconsistencies that can be used to refine any manufacturing process. Another enabling technology, namely DT permits for virtual testing and optimization of a manufacturing process, resulting in enhanced productivity and reduced costs [32]. Sensors play a critical role in DTs as well in the near-real-time data collection and its utilization for the continuous improvement of DT, ensuring that the DT model precisely mimics physical assets [33]. It ultimately aids in rapidly updating decisions, adjusting process or machine variables, and ultimately enhancing the efficiency of the manufacturing process by lining up the DT model with its physical counterpart [34]. The benefits of D^2^F are evident; nevertheless, its implementation and performance require demonstration and validation to ensure that the interconnection of digital technologies is seamless and bolsters the anticipated productivity. To address these challenges, this review article compiles 5 sections. Section 2 highlights FPGAs and traditional sensing devices (SDs) and their adaptability to D^2^F operations, Section 3 investigates the effect of FGPA-based SDs in DTs, Section 4 details the effect of FPGA-based SDs for sustainable D^2^F operations, and Section 5 discusses the conclusion and future outlook.

## 2. Sensing Devices

FPGA-based sensing devices are still relatively new in manufacturing due to the inherent intricacy, the specific skill sets needed, and the complexity involved in an efficient design and its implementation. A few researchers tried to adopt this technology for various manufacturing processes, as shown in Table 1; however, the implementation is nascent.

**Table 1:** Application of FPGAs in manufacturing processes.

Manufacturing Process	Feature Monitored	References
Additive manufacturing	Defects inspection	[35]
Milling process	Failure monitoring	[36]
Hybrid additive manufacturing	In-line process monitoring	[37]
High speed face milling process	Tool condition monitoring	[38]
Grinding process	Real-time process monitoring	[39]
Laser additive manufacturing	Control system to monitor and control the process parameters	[40]

The design procedures for FPGA-based SDs are complex, demanding extensive comprehension of hardware modeling languages and system interfacing, preventing widespread adoption. Besides, the expenditure of FPGAs over conventional SDs may not warrant their use for all applications. Inadequate research penetration of FPGAs in relation to manufacturing processes further promotes their sluggish adoption. Due to the dearth of literature related to FPGA adoption within manufacturing, this section will discuss FPGA fundamentals and the various sensors developed using FPGA and their applications. Lastly, the extension of FPGA to manufacturing processes within D^2^F will be discussed.

### 2.1. FPGA-Based Sensing Devices

**Figure 1:**
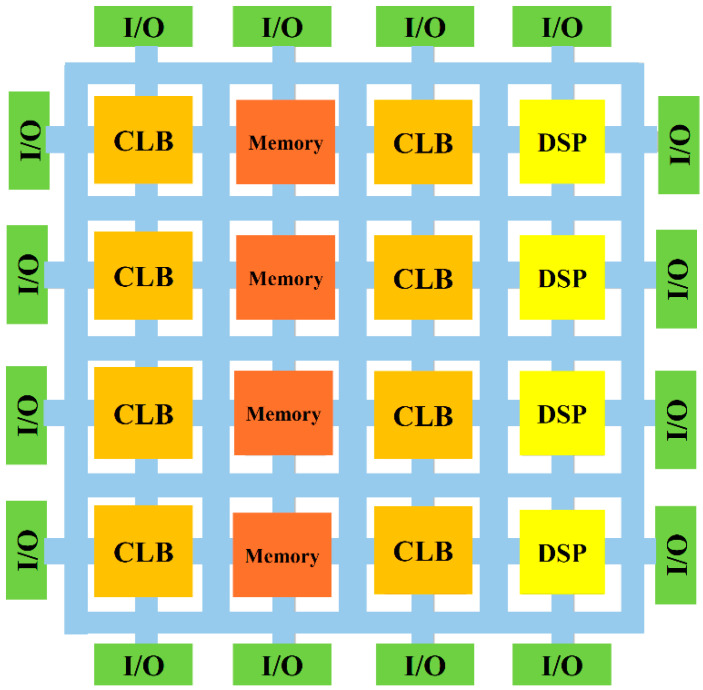
A schematic architecture of FPGA, based on the data provided in Ref. [46]; CLB, DSP, and I/O are abbreviated as configurable logic blocks, digital signal processor, and input/output, respectively.

Figure 1 shows the schematic architecture of an FPGA. FPGAs are made up of multiple building blocks. By combining these building blocks, users can create a vast array of digital circuits and systems. The main FPGA components and their fundamental roles are listed below.

- Configurable Logic Blocks (CLBs)

CLBs are a type of logic block. Logic blocks are the foundation of any FPGA that are made up of multiplexers, flip-flops, and look-up tables (LUTs). Their duties include implementing sequential logic (with flip-flops) and combinational logic (with LUTs). These blocks perform basic logic functions (e.g., AND, OR, XOR) and are combined to create more complex digital circuits.

- Blocks for input/output (I/O) Interface:

I/O blocks serve as an interface between the external environment (e.g., sensors, other devices, or a CPU) and the internal FPGA circuitry. Data transfer communication protocols and voltage-level conversion of electrical signals are handled by these blocks.

- Digital Signal Processing (DSP)

These specialized blocks are crucial for signal processing tasks because they are made to perform high-speed arithmetic operations like multiplication, addition, and accumulation. They are frequently utilized in applications that need to process data in real-time, like filtering and processing audio or video.

- Memory blocks

Block RAMs are used to store data or instructions during operation. BRAM modules offer specific memory storage within the FPGA. They are typically used for temporary data storage, buffers, or implementing small memory-intensive operations.

Sensor nodes mostly utilize digital signal processors (DSPs) to process data. FPGA-based sensing devices enable a specific hardware technology that is also reprogrammable, yielding a reconfigurable sensing system. Partial reconfiguration is a technique of altering only specific pieces of logic incorporated in an FPGA [41], resulting in a circuit that can be adjusted to perform various functions. It, in-return, enables the construction of complicated applications by employing dynamic reconfigurability at low power utilization [42]. This is useful for current sensing devices (SDs) when required to transfer high accuracy and resolution data with higher precision while reducing area and power consumption. FPGAs and their inherited feature of reconfigurability enable the addition of processing capacities, interfaces, analyzes modules, and configuration to SDs [43]. FPGA-based sensors are also designated as smart sensors. FPGAs capabilities also enable the creation of not just logic and state-based circuits, but also high-level soft processors [44]. FPGA-based logic components are used in FPGA-based embedded processors to create internal storage units, data and control buses, as well as on-chip and off-chip peripheral and memory controllers [45].

FPGA-based processors can be divided into (a) hard and (b) soft processors. Table 2 details the pros and cons of both categories.

The hardware resources in FPGAs vary widely depending on the producer (different manufacturers) and the individual FPGA platform. Nevertheless, a large variety of devices incorporate components like Sensors, Processors, Flip Flops, Communication Modules, I/O Interfaces and Power Management Units, making them suitable for SDs. Most FPGAs could support adjustable input and output standards, allowing a variety of devices to be linked and function at varying voltage levels without the necessity for voltage converters, significantly streamlining design and reducing costs [47]. For instance, in the case of the “Xilinx’s Spartan-3”, a variety of input and output standards are supported with the operating voltage between 1.20 V to 3.30 V, including Low-Voltage CMOS, Low-Voltage TTL, Gunning Transceiver Logic, High-Speed Transceiver Logic, Peripheral Component Interconnect, Stub Series Terminated Logic, Lightning Data Transport or Hyper Transport, Low-Voltage Differential Signaling, Reduced Swing Differential Signaling, and Low-Voltage Positive Emitter-Coupled Logic [48]. Some FPGAs have a significant number of “arithmetic blocks”, ranging in complexity from basic multipliers to DSP units, and are sometimes a combination of multipliers, accumulators, adders or shift registers [49]. A DSP unit is responsible for an enhanced working performance of FPGA, permitting elevated output and flexibility at the expense of cost and energy utilization. For example, Altera’s Stratix II and GX devices have multiple DSP columns that can rapidly execute multiple operations [50].

**Figure 2:**
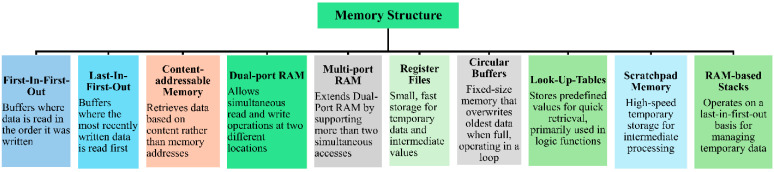
Memory structures in FPGA; based on the data provided in Refs. [53,54,55].

Internal memories (IMs) modules are far faster than external memories which is one of the major advantages of FPGAs. It is important to clarify that this likely refers to Block RAM, which operates in the MHz range, as opposed to the GHz speeds of DDR4 memory. IMs are physically located within the FPGA and are close to the programmable logic elements. This minimizes the delay caused by data transfer, as there are no external interfaces. On the other hand, External DDR memories are present outside the FPGA, requiring communication using memory interfaces such as DDR controllers, which introduce latency. For instance, Xilinx Virtex UltraScale+ series provides up to 500.0 Mb of IM, while Intel Stratix 10 delivers up to 229.0 Mb of IM [51,52]. These memories contain different types of RAMs, including block RAM, Ultra RAM, and distributed RAM, permitting a competent handling of large volume datasets. This allows for high-speed management and processing in even the most demanding applications, including AI/ML, real-time data analytics, and 5G connections. This expansion in IM improves the execution and flexibility of FPGAs, making them ideal candidates for intricate and data-intensive jobs. FPGAs can be implemented in a variety of memory structures as provided in Figure 2.

FPGAs lower cost, expanding capabilities, and the ability to improve the performance of SDs with particular hardware technologies have resulted in a significant surge in their employment in new sensor-related applications. There are various applications that require high data throughput, including real-time image and video processing, signal processing, and wireless communication systems, and can benefit from the emerging chip density [56]. These sensing systems and devices based on FPGA have been elaborated in the following sub-sections.

#### 2.1.1. Vision-Based FPGA Control Systems

Vision-based information can benefit from FPGAs’ parallel processing capabilities [57,58]. A neuro-inspired mobile robot with a double spike-based control system for two direct control motors was proposed in Ref. [44]. Here, an FPGA was used to handle all image processing information. A similar strategy was described in Ref. [59], utilizing an event-driven representation for sensing, dealing, and starting a robot. Besides, a hardware and software design and execution plan for robot localization was also proposed [60]. This work, particularly designed for the Mars rover mission, utilized a system architecture developed on a “Xilinx Virtex-6” FPGA to process acquired photos, carry out visual-based localization and mapping, and three-dimensional reconstruction, to determine the rover’s position. Other researchers have demonstrated an automated approach for evaluating liquid stages in membrane distillation processes [61]. This technique was established on the “laser triangulation” method, having two lasers and a camera, along with the FPGA to process all the acquired images. In Ref. [62], the “simple network robot protocol” was applied to combine network robots with sensing devices. For this purpose, an FPGA was uttilized to create a real-time image processing system that delivers “simple network robot protocol” provisions. The proposed module was implemented as “visual servoing” technique for industrialized robots.

Although they have been adopted in wide-ranging, demanding situations as discussed, vision-based FPGA applications are very limited in distributed manufacturing ecosystem [63]. The authors of this current study consider that the integration of vision-based FPGA sensing devices will perform a vital role in augmenting the working efficacy and precision of D^2^F by incorporating the high-speed image processing abilities inherited by FPGAs equipped with the parallel processing. Such devices will result in real-time monitoring and scrutiny of a manufacturing process via sophisticated computer vision methods, allowing for quick recognition and classification of abnormalities, flaws, and variations in a manufacturing process. The use of FPGAs in vision systems enables the realization of customized and efficient processing algorithms that can process substantial amount of data with the least latency, resulting in better process and quality control, as well as timely decision making within D^2^F environment. These devices will also contribute to the robustness and flexibility of DTs and smart manufacturing by supporting flawless data acquirement and handling, thus optimizing the system operation for higher efficiencies and lowering interruptions.

#### 2.1.2. Process Monitoring and Control Using FPGA Sensing Devices

Various applications not only require sensing data to be acquired, but also that the data to be processed so that comparison can be done against a reference. Based on this criterion, a framework has been proposed in Ref. [64] by utilizing an FPGA-based monitoring infrastructure. As an example, a computer vision system for liquid level sensing in membrane distillation applications is provided in [61]. In another study, process monitoring and control was proposed using FPGA. The framework utilized a CMOS camera along with FPGA-based computer vision processor. The proposed FPGA framework was able to measure the levels of liquid with an accuracy of 0.013 mm in hot and cold reservoirs. In another instance, a novel thermal monitoring system was developed using FPGA with JBit technology. Various fast and low-cost experiments were carried out by leveraging the benefits of the rapid design cycles and re-configurability of FPGAs, and this method was employed to establish a thermal map within a die.

In a D^2^F, the latency period for feedback is a vital factor that absolutely affects the system’s working performance and efficacy. Low latency is necessary to guarantee real-time harmonization and uniform interaction between several interconnected systems. Delays in response times can cause substantial disturbances, compromising the system response, leading to variations in anticipated product quality. Hence, optimizing the feedback latency is of utmost importance for retaining the reliability and trustworthiness of any D^2^F ecosystem while demonstrating a deterministic advantage of TFs. In such cases, FPGAs can play a critical role in reducing these feedback delays in manufacturing.

#### 2.1.3. FPGA-Based Advanced Control Systems: Predictive, Fuzzy, and Neural Network-Based Controllers

**Figure 3:**
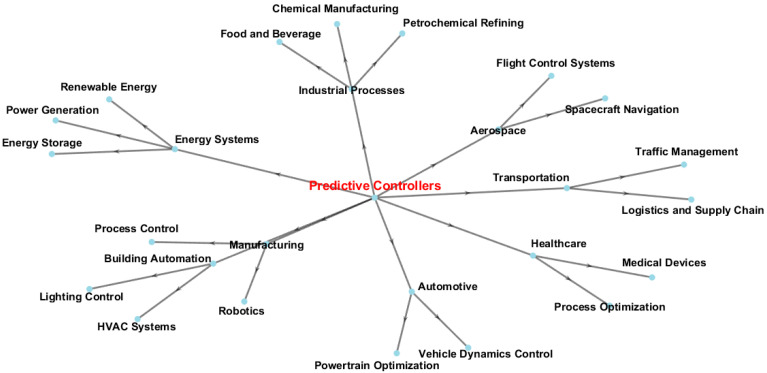
Predictive Controllers applications, based on the data provided in Refs. [66,67,68,69].

FPGAs have recently been utilized to create various control systems, such as fuzzy logic systems (FLSs), neural networks (NNs), and predictive controllers (PCs) [65]. PCs provide a recognized control approach that can be applied in various areas. The design of the FPGAs is ideally suited to these controllers’ parallel nature and has been implemented to measure variables like thermal distribution and ambient conditions. Figure 3 demonstrates the applications of PCs in various fields of life.

FLSs controllers are another type of control system that has been applied with FPGAs [70,71,72]. These frameworks may be built using FPGA coupled with the control technique defined by Fuzzy logic rules; thus, negating the need for extensive modeling. These FPGAs can process the fuzzy algorithms’ high workload to attain high levels of precision in real-time. A model-based design technique was explored for realizing fuzzy logic controllers on FPGA [73].

NNs, known for their capacity to understand and characterize complicated, non-linear systems, offer substantial advantages in control applications where traditional approaches fail. NNs in combination with FPGA’s parallel processing abilities may result in high-speed as well as real-time processing. This sequence facilitates the employment of highly effective and reactive control systems, to drive critical and rapid decision-making applications. In one recent study, an FGPA-based accelerator was proposed to deal with the performance deficiency of general-purpose processor in the case of convolutional NNs [74]. It was identified that the FGPA-based solution resulted in 16.42 times faster results compared to the traditional processor. FPGAs facilitate hardware customization to converge into the special requirements of NNs, permitting optimized resource consumption and power efficacy. This is exceptionally helpful in circumstances where the control system must constantly understand and adapt to dynamically varying situations. The FPGA’s reconfigurability also establishes reiteration, assisting updates to the NN’s architecture without expensive hardware redesigns.

**Figure 4:**
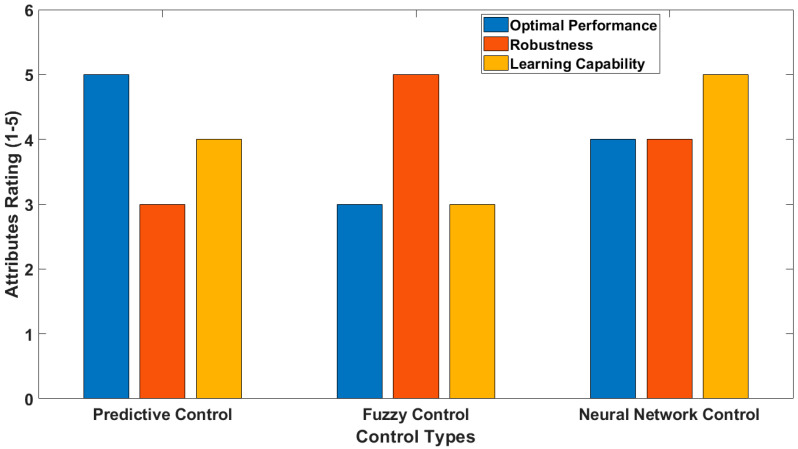
A comparative analysis of FPGA-based advanced predictive, fuzzy, and neural network control systems.

Many AM processes yield internal defects, requiring real-time assessments with the potential for rectification to fix the defects during layer-by-layer processing. In a recent analysis, a defect database (NEU-DET) was utilized to train convolutional NNs for defect classification [75]. The real-time inspection was carried out using FPGAs and a tuned and binarized neural network (BNN). The framework also employed selective search and non-maximum suppression techniques to identify defects. The results demonstrated that the BNN model was able to detect faulty photos with a 97.9% accuracy within 0.50 s. Figure 4 provides a comparison overview of FPGA-based advanced predictive, fuzzy, and neural network control systems.

Similarly, FPGA-based advanced control systems will provide substantial advantages in a D^2^F environment. These controllers will result in real-time, rapid decision-making and accurate control of complicated and connected processes within a factory or a geographically distributed group of factories. PCs can be leveraged to predict upcoming system conditions, adjust manufacturing schedules, and lower idle time, while FLSs can be used to specify robust control over imprecise data, which is necessary in dynamic manufacturing circumstances. NNs-based controllers will provide adaptive learning, granting the system to endlessly iterate and improve its working performance by evaluating the sensing data. The parallel processing abilities and reconfigurability of FPGAs confirm these controllers will work efficiently, controlling the D^2^F environment where the manufacturing processes coordinate with each other seamlessly.

#### 2.1.4. FPGA-Based Digital Signal Processing

FPGAs also provide embedded signal processing (SP), which is a point of interest for various applications. Prior to FPGAs, DSPs were the primary candidate for signal-processing devices. For extreme applications, FPGAs have now exceeded DSPs owing to the efficacy of their parallel processing, on-chip memory, reconfiguration, and algorithm development performance [76,77,78].

FPGAs have been utilized to realize these kinds of sensor data processing. A prototype was developed to transform the traditional pulse Oximeter into a wireless sensing device using FPGA [66]. The device was linked to a XBee wireless modem. Using the preset protocols to transfer data from the local to central servers, the system was able to produce alarms when the vital signs were critical. In Ref. [79], spike-based bandpass filters were created using FPGAs. These filters were able to deny out-of-band frequency within the spike realm. This framework yielded advantages to execute extreme parallel processing exclusive of intricate hardware, allowing the incorporation of elevated filter numbers within the FPGA. In other cases, FPGAs have also been applied to process ultrasonic signals. These signals are subjected to low-level processing, and the FPGA used improved scanning rates, precision, and reliability [80]. Such operations are necessary to perform high level processing, including feature extraction and analysis. A reconfigurable FPGA-based system was proposed for ultrasound signal processing, enabling flexible beamforming and realizing real-time operation for advanced imaging methods. In another study [81], a multichannel FPGA-based random waveform producer was presented, advancing flexibility in ultrasound signal equipped with elevated speed and resolution abilities. Furthermore, a near-real-time three-dimensional beam-former and scan converting system was proposed on FPGAs, attaining expressive speed enhancements by processing 30 volumes/s over conventional systems and allowing real-time three-dimensional ultrasound signal process [82]. These studies prove that FPGA employments in ultrasonic signal processing have provided considerable enhancements in system flexibility, performance speed, and real-time abilities, empowering advanced imaging methods for an efficient analysis for different applications.

As detailed through the research examples in this section, FPGA-based signal processing is critical for a D^2^F as it allows for real-time processing of large volume datasets stipulated by the need for these sensing devices within the factory. FPGAs are programmable and allow to execute complicated signal processing functions such as signal data filtering and conditioning at the source, reducing the necessity for consolidated processing and reducing communication latency. This approach upgrades the factory’s capability to adjust promptly to altering operational conditions, improves the system’s reliability, and permits for additional effective administration of manufacturing operations. Besides, FPGAs’ parallel processing ability makes them an ideal candidate for organizing dynamic data streams within a distributed digital factory environment, allowing for the structured implementation of advanced automation and intelligent production efforts.

#### 2.1.5. FPGA-Based Sensor Array

A sensor array (SA) is a pool of sensing nodes (SNs) connected to a network and dispersed within a region of interest and employed to examine physical as well as environmental factors [83]. These devices have restrictions with respect to computing capacity, connection, and energy utilization. Currently, the number of applications utilizing such devices has grown substantially in environmental studies, image processing, construction, agriculture, and many other applications [84,85,86]. The applications mentioned above require reduced power since the SNs are low-cost, and work in circumstances with limited computational capacity and battery life. Low-energy SAs can be deployed in engineering areas to safeguard and prolong the realistic lifespan of most products. For such applications, FPGAs allow considerable increase in energy efficacy owing to their effective utilization of communication networks. Additionally, the FPGAs operate as decentralized adaptive systems, granting the creation of various functionalities using long-distance resources. For such applications, various researchers have worked to use the FPGAs to lessen data transfer among SNs [87,88], alter the adaptive frequency [89,90], and activate radio transceivers as needed [91,92].

The authors in Ref. [93] provided a scattered framework for combining micro-electromechanical systems (MEMS) via a layered communication architecture controlled by a central node. The micro-electromechanical system links sensors with signal processing and communication modules, yielding reduced size, weight, and energy utilization. For this purpose, the authors created an interface linking micro-electromechanical systems to a sensor. This interface was designed in VHSIC hardware description language (VHDL), and the subsequent core was integrated into the designed micro-electromechanical system. The resultant core includes an interface file system, offering all vital facts about the developed system that can be connected to a network. The architecture was successfully developed and tested on a FPGA, resulting in small, versatile, customizable, and reconfigurable nodes.

Another use of FPGA is to lessen the energy consumption in an effective communication network. In Ref. [94], FPGA was applied to realize the less energy utilization of a Wake-up Radio for wireless network communication. To reduce energy consumption, various schemes were applied. However, the “clock gating” scheme was most successful in reducing power consumption. This scheme disables a specific block if not used while the static energy consumption remains unchanged. The results fell within the Cost-of-Test performance range, which is highly competitive given the abilities demonstrated using an ultra-low-power FPGA.

The authors in Ref. [95] established an innovative infrastructure for data-rich and high-capacity computation using FPGAs. Combining ubiquitous dynamic resources within a grid model resulted in a reconfigurable platform for the synchronized and distributed utility of heterogeneous FPGAs. This technique offered significant benefits in a system design through locality and access transparency, as well as the remote triggering of reconfiguration tasks. Especially in power-restricted conditions, this technique enables the long-distance management and configuration of low-power and cost-effective FPGAs. Various other researchers also explored the utilization of FPGA in SNs and mobile communication. A quick evaluation algorithm was proposed for mobile sensing devices incorporated with heterogeneous FPGAs, adjusting weights to improve the Quality of Service in Ref. [96]. Others have proposed an adaptable sensing network utilizing FPGAs for delay-based evaluation, highlighting their flexibility in hardware design for diverse situations [97]. FPGAs can also be helpful in quick prototyping and lowering redesign attempts.

FPGA-based SAs present noteworthy benefits to the D^2^F network by increasing real-time data administration and transmission efficacy. These arrays allow localized calculation, allowing for speedy investigation and reaction to the assimilated data, which is necessary for providing best factory performance and reducing latency. Their capability to be modified and reconfigured indicates that they can be amended to particular factory requirements. Additionally, FPGAs influence energy efficacy by adjusting power use via techniques such as asynchronous designs or purpose-specific designs that efficiently map to sensing or computation requirements, lowering energy requirements and widening the operational duration of sensing devices on the network. Additionally, FPGAs influence energy efficacy by adjusting power use via techniques such as asynchronous designs or purpose-specific designs that efficiently map to sensing or computation requirements, lowering energy requirements and widening the operational duration of sensing devices on the network. By eliminating global clocks and allowing activity-based power consumption, asynchronous logic holds promise for lowering dynamic power. Using customized designs that make use of hard IPs, DSP slices, and BRAMs, domain-specific FPGA applications—like sensor networks, signal processing, and machine learning—achieve notable power savings by minimizing off-chip memory accesses and streamlining data pathways. FPGA-specific optimizations, such as dynamic partial reconfiguration, multi-clock domain management, and power-aware routing, provide more granular and application-driven power savings than traditional methods like clock gating, which is less effective in FPGAs because of routing and timing problems. These FPGA advantages of real-time handling, customization, and power efficiency facilitate the robustness of D^2^F.

#### 2.1.6. FPGA-Based Intelligent Sensors

The requirement for compact, precise, and power efficient sensing devices has risen recently. Here, intelligent sensing devices (ISDs) are referred to devices that unite various functions into a single unit, and perform actions such as data transmission, diagnostics, decision making, and various autonomous functions. When discussing ISDs, the integration of these functions into an FPGA-based system is common. FPGAs, with their adaptability, enable the integration of different features, including signal manipulation and analysis [98,99,100]. Moreover, ISDs can perform various other functions besides data collection such as error correction or derive intricate information from measurements. An example of the aforementioned is the derivation of resistivity and capacitance from sensory data, or the addition of features to thermal sensing data via FPGAs [101,102,103,104]. Furthermore, the term intelligent camera is utilized for cameras that combine integrated video capture, processing, and transmission into a single embedded system [105]. Here, the integration of hardware and software has been carried out using an FPGA. FPGAs also allow to process the large size and volumes of image data, permitting the amalgamation of low-tier and medium-level vision algorithms [106]. In this setup, the intelligent camera not only captures images but also processes the data, becoming an ideal candidate for data-intensive purposes, an example including the handling of touch-based sensing data [107].

The use of ISDs and intelligent cameras along with FPGA will be beneficial for D^2^F. ISDs combine numerous functions into a single unit, streamlining functions and improving effectiveness. FPGAs present flexibility for signal handling and fault adjustment, bolstering the precision of sensor data. Intelligent cameras can process and evaluate a huge dataset of images and are ideal candidates for quality monitoring and control. These advanced data processing techniques can be leveraged by D^2^F to enhance their performance. Table 3 summarizes all the studies for FPGA sensing devices.

**Table 3:** Overview of FPGA-based sensing and controlling devices as discussed above.

FPGA Implementation Domains	Outline	Research Domain	Outcomes	Ref.
Vision-based FPGA Control Systems	Image processing and control in robotics and industrial applications	Neuro-inspired mobile robot	Proposed a double spike-based control system for two direct control motors using FPGA	[58]
		Event-driven robot representation	Employ an event-driven interpretation for sensing, processing, and starting a robot	[59]
		Robot localization for Mars rover	Elaborated a system architecture on the Xilinx Virtex-6 FPGA for visual-based localization, mapping, 3D reconstruction, and position identification	[60]
		Liquid level evaluation in membrane distillation	Proposed an automated approach using laser triangulation with two lasers, a camera, and an FPGA	[61]
		Network robots with sensing devices	Employed simple network robot protocol to integrate network robots with sensing devices, using FPGA	[62]
Process Monitoring and Control Using FPGA Sensing Devices	FPGAs in monitoring and control applications	Monitoring infrastructure	Proposed a framework for process monitoring and control	[64]
		Thermal monitoring system	Developed a novel system using FPGA with JBit technology for fast, low-cost experiments and establishing thermal maps within a die	[65]
FPGA-based Advanced Control Systems	Implementation of predictive, fuzzy, and neural network-based controllers on FPGAs	Accelerator for convolutional NNs	Achieved 16.42 times faster results compared to traditional processors	[74]
		Real-time defect detection in AM	Used FPGA and binarized NN for defect classification, achieving 97.9% detection	[75]
FPGA-based Digital Signal Processing	FPGAs in signal processing	Wireless pulse oximeter	Transformed traditional pulse oximeter into a wireless sensing device	[108]
		Spike-based bandpass filters	Created filters using FPGA to deny out-of-band frequency	[79]
		Ultrasound signal processing	Better scanning rate, precision, and reliability	[80]
		Reconfigurable ultrasound signal processing	Enabled flexible beam-forming and real-time operation	[81]
		3D ultrasound beam-former	Near-real-time 3D beamforming and scan converting, processing 30 volumes/s	[82]
FPGA-based Sensor Array	FPGAs in sensor networks	Micro-electromechanical systems integration	Developed a scattered framework with layered communication architecture	[93]
		Wake-up Radio for wireless network	Applied clock gating scheme to reduce power consumption	[94]
		Data-rich computation infrastructure	Established a reconfigurable platform for synchronized and distributed heterogeneous FPGAs	[95]
		Mobile sensing device evaluation	Proposed a quick evaluation algorithm for devices	[96]
		Adaptable sensing network	Proposed network using FPGAs for delay-based evaluation	[97]
FPGA-based Intelligent Sensors	Integration of multiple functions into single sensor	Intelligent cameras	Integrated video capture, processing, and transmission in a single system	[106,107]
		Touch-based sensing data handling	Used intelligent cameras with FPGAs for processing data-intensive touch-based sensing	[107]

### 2.2. Traditional Sensing Devices Utilized for Manufacturing Processes

Many sensing devices have been used to monitor the manufacturing process in near-real-time, which are important for demonstrating and assuring the quality of a manufactured product [109,110]. In advanced manufacturing processes such as AM, the manufacturing of a part is carried out in a layer-by-layer fashion, and the characteristics of each layer can ultimately define the final part quality. Conversely, in MRPs, a final product is manufactured by machining material from a solid block [111]. The commonly used machining processes are electric discharge machining, milling, turning, and drilling. The processing conditions in both AM and SM processes have a significant effect on surface roughness, part dimensional accuracy, mechanical and tribological properties of the manufactured product [112]. Therefore, process monitoring devices are usually applied to monitor various processing conditions during manufacture such as thermal distribution, pressure, humidity, meltpool dimensions, reactive forces and many other variables of importance [113,114]. The information from these sensing devices are then utilized to optimize the manufacturing process [115]. Various researchers are working to modify the process parameters in real-time to minimize process-induced defects, particularly in the case of AM processes [116]. These defects include porosity, delamination, and residual stresses, providing products with enhanced properties [117]. The list of process-induced defects, and monitored using traditional sensing devices is provided in [118]. Table 4 compiles the defects generated in laser AM and MRPs.

**Table 4:** Monitored defects in laser AM and MRPs.

Process	Defects	Description	Ref.
**Laser AM**	Porosity, balling, and lack of fusion	Due to poor fusion, powder spatter, and non-unform melt pool formation	[119,120,121]
	Residual Stresses	Due to rapid cooling/heating	[122]
	Distortion	Geometry changes due to thermal effects	[123,124]
	Surface morphology	Variations in surface texture	[125]
	Heterogeneous microstructure	Inconsistent microstructural features	[126]
	Crack formation	Due to induced thermal stresses	[127]
	Geometrical anomalies	Unexpected shape deviations	[128]
	Thermal gradient imbalance	Non-uniform cooling	[129]
**MRPs**	Surface Roughness	Irregularities on machined surfaces	[130]
	Dimensional Inaccuracy	Deviation from design dimensions	[131]
	Burr Formation	Small edges left after cutting	[132]
	Thermal Damage	Heat-affected zones during machining	[133]
	Tool Wear	Gradual loss of tool material	[134]
	Surface Cracks	Microcracks due to fatigue or stress	[135]
	Chatter Marks	Vibrational marks on surface	[136]
	Overheating	Excessive heat during cutting	[137]
	Fatigue Cracks	Cracks from cyclic stress	[138]

**Figure 5:**
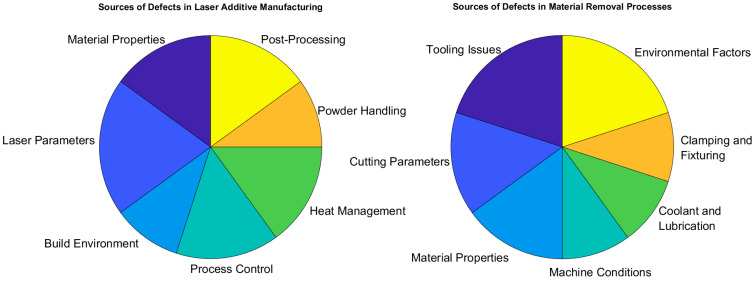
Sources of defects in laser AM and material removal processes; based on the data provided in Refs. [139,140].

The primary sources of the induced defects have been provided in Figure 5. Defects in laser AM and MRPs can be from various sources. For laser AM, defects include discrepancies in thermo-physical properties, non-optical laser beam settings, unstable build conditions, non-optimized process controls, inaccurate thermal management, and feedstock characteristics, as well as post-processing conditions, which can all cause defects such as porosity, warping, and part distortion. In MRPs, flaws are usually the result of tooling, non-optimal cutting parameters, machine equipment conditions, material properties, non-optimal coolant, material clamping issues, and ambient conditions. Hence, it is crucial to understand and minimize such defects, which will lead to high-quality parts.

#### 2.2.1. Traditional Sensing Devices Used for Laser AM Processes

**Figure 6:**
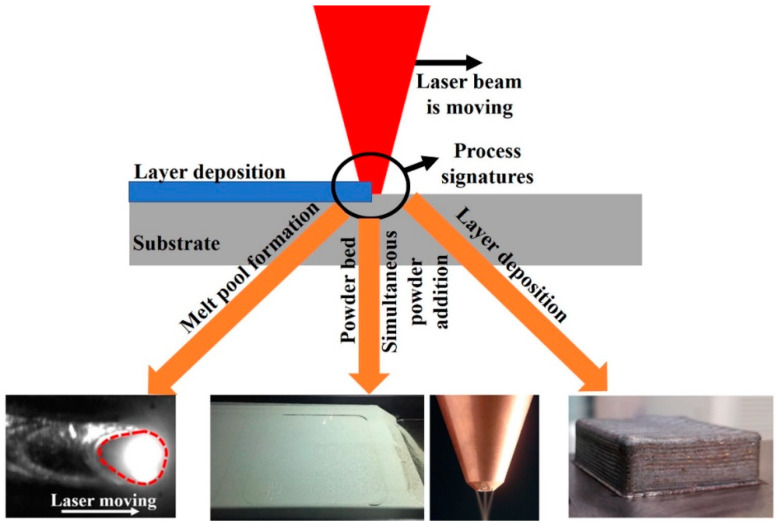
Features measured in laser AM processes [141]; published under open-access license.

The materials usually exhibit distinct signals during laser AM that can be assessed to examine AM process parameters. These signals are also known as process signatures and can explain melt pool characteristics, deposited layer characteristics, and part properties. These process signatures can be controlled within an ideal regime to assure high-quality printing, as depicted in Figure 6 [141]. The sensing devices are useful in monitoring these ideal processing conditions and can be utilized to optimize the processing conditions, yielding parts with excellent mechanical performance and dimensional qualities.

**Figure 7:**
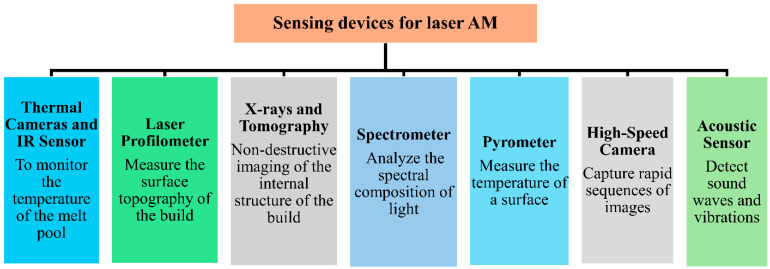
Features measured in laser AM processes [141]; published under open-access license.

Figure 7 compiles the major classification of traditional sensing devices used to monitor laser AM processes. They include thermal and IR cameras, laser profilometry, X-rays and X-ray computed tomography, spectrometry, pyrometry, high-speed imaging cameras, and acoustic sensors. The data collected from these devices is subjected to further processing to ascertain desired quality metrics about the process [142]. The degree of required information allows for the determination of the sensing device.

Table 5 compiles the various features measured using traditional sensing devices applied to monitor the laser AM process.

**Table 5:** Features measurement in laser AM processes, based on the data provided in Ref. [141] that is published under an open-access license.

Feature	Material	Refs.
**Molten pool**	-	[143]
	Ti-alloy	[144]
	Ti-alloy	[145]
	Ti-alloy	[146]
	-	[147]
	AlSi10Mg	[148]
	-	[143]
	-	[149]
	Ti-alloy	[150]
	SS 316L	[151]
	AlSi10Mg	[148]
	-	[143]
	SS 304L	[152]
	-	[149]
	SS 304L	[153]
	SS 316L	[154]
	Ti-alloy	[146]
	Ti-alloy	[150]
	AlSi10Mg	[148]
	SS 304L	[153]
	IN-625	[155]
	SS 303L	[156]
	SS303L	[157]
	Ni-55	[158]
	H13	[159]
	H13	[160]
	-	[161]
	SS 303L	[162]
	SS 42C	[163]
	IN-718	[164]
	IN-625	[165]
	SS 316L	[166]
	SS 42C	[167]
	Ti-alloy	[168]
	IN-718	[169]
**Layer printing**	SS 316L	[170]
	SS 316L	[151]
	SS 904L	[171]
	IN-718	[172]
	SS 304A	[173]
	IN-718	[174]
	IN-625	[175]
	IN-625	[176]
	-	[177]
	SS 316L	[178]
	Mo-W	[179]
	IN-625	[180]
	IN-625	[176]
	-	[177]
	HX	[181]
	Ni-alloy	[182]
	IN-625	[180]
	SS 316L	[183]
	SS 42C	[184]
	C-Mn steel	[185]
	SS 316L and Ti-alloy	[186]
	SS 303L	[187]
	Ti-alloy	[188]
	Ti-alloy	[189]
	Ti-alloy	[190]
	SS 316L	[191]
	H13	[192]
	Ti-alloy	[193]
**Feedstock**	SS	[194]
	-	[195]
	-	[196]
	Ti-alloy	[197]
	-	[198]
	IN-625	[176]
	IN-625	[180]
	HX	[181]
	-	[195]
	Ti-alloy	[197]
	Ti-alloy	[199]
	SS 904L	[171]
	IN-625	[155]
	Ti-alloy	[200]
	SS 316L	[201]
	Ni-Cr-B/Tungsten carbide composite	[202]

Table 6 collects the various traditional sensing devices applied to monitor laser AM process.

**Table 6:** Sensing devices used in laser AM processes, based on the data provided in [142] that is published under an open-access license.

Feature	Material	Sensing Device	Reference	Feature	Material	Sensing Device	Reference
**Molten pool**	Ti-alloy	PT	[144]	**Layer printing**	SS 316L	ASD	[170]
	Ti-alloy	PT	[145]		SS 316L	THC	[151]
	-	IRC	[143]		SS 904L	PT	[171]
	Ti-alloy	PT	[146]		IN-718	THC	[172]
	AlSi10Mg	IRC	[148]		SS 304A	THC	[173]
	-	IRC	[143]		IN-718	THC	[174]
	-	IRC	[149]		IN-625	THC	[175]
	SS 316L	TIC	[151]		IN-625	IRC	[176]
	AlSi10Mg	IRC	[148]		-	IRC	[177]
	-	IRC	[143]		SS 316L	ASD	[178]
	SS 304L	IRC	[152]		Mo and W	THC	[179]
	-	IRC	[149]		HX	IRC	[181]
	SS 304L	PT	[153]		Ni-alloy	IRC	[182]
	SS 316L	IRC	[154]		IN-625	IRC	[180]
	Ti-alloy	PT	[146]		SS 316L	VRC	[183]
	Ti-alloy	IRC	[150]		SS 42C	VRC	[184]
	AlSi10Mg	IRC	[148]		C-Mn Steel	VRC	[185]
	SS 304L	THC	[153]		SS 316L and Ti-alloy	VRC	[186]
	IN-625	IRC	[155]		Steel with low carbon	VRC	[203]
	SS 303L	VRC	[156]		SS 303L	VRC	[187]
	SS 303L	VRC	[157]		Mild steel	VRC	[204]
	Ni 55	IRC	[158]		Ti-alloy	VRC	[188]
	H13	IRC	[159]		Ti-alloy	VRC	[189]
	H13	IRC	[160]		Ti-alloy	VRC	[190]
	-	VRC	[161]		SS316L	ASD	[191]
	SS 303L	VRC	[162]		Ti and Ni alloys	ASD	[205]
	SS 42C	VRC	[163]		H13	XYs	[192]
	IN-718	IRC	[164]		Ti-alloy	XYs	[193]
	IN-625	ASD	[165]				
	SS 316L	PTE	[166]				
	42C steel	PTE	[167]				
	Ti6Al4V	ASD	[168]				
	IN-718	VRC	[169]				
**Feedstock**	SS	PT	[194]				
	-	PT	[195]				
	-	THC	[196]				
	Ti-alloy	THC	[197]				
	IN-625	IRC	[176]				
	IN-625	IRC	[180]				
	HX	IRC	[181]				
	IN-625	IRC	[155]				
	Ti-alloy	IRC	[200]				
	-	PT	[195]				
	Ti-alloy	THC	[197]				
	Ti-alloy	THC	[199]				
	SS 904L	THC	[171]				
	-	THC	[198]				
	SS 316L	ASD	[201]				
	Ni-Cr-B/Tungsten carbide composite	IRC	[202]				

Note: Visible range camera = VRC, IR camera = IRC; Thermal camera = THC, Acoustic-based sensing device = ASD, Pyrometer = PT, Photodiode = PTE, X-rays = XYs.

#### 2.2.2. Traditional Sensing Devices Used for Material Removal Processes

Like laser AM processes, material removal processes (MRPs) also use various sensing devices (SDs) to examine the machining process [206,207,208]. These SDs play a key role in confirming accuracy, monitoring tool life, improving surface outlines, and reducing manufacturing cost by monitoring real-time data [209,210,211]. Table 7 collects the various sensing devices that have been used in MRPs.

**Table 7:** Sensing devices used in manufacturing processes.

Sensors	Functionality	Applications	Ref.
Force measurement sensing device	Cutting forces analysis	Traditional manufacturing processes such as milling and drilling	[212]
Acoustic-based sensing device	High-frequency signal measurement for cracks	Tool failure analysis	[213]
Vibration sensing device	Vibration in tool and working piece	Chatter identification	[214]
Temperature sensing device	Temperature analysis within the cutting zone	Tool wear and workpiece temperature management	[215]
Tool wear sensing rate	Tool wear measurement	Prognostic maintenance	[216]
Energy sensing device	Measure power consumption	Energy efficacy	[217]
Pressure sensing device	Detect flow of cutting fluid	Cooling system optimization	[218]
Strain measurement device	Identify deformation in machine elements	Machine health checking	[219]

Table 8 presents the various studies from the literature on the parameters measured using the specific sensing devices.

**Table 8:** Parameters measured using various sensors.

Parameters Measured	Sensing Device Type	Ref.
Surface finish, tool wear, vibration, cutting forces, speed	Dynamometer, acoustic sensor, temperature sensor	[220]
Cooling fluid consumption	Dynamometer	[221]
Cutting forces, feed rate	Force sensor	[222]
Cutting forces	Force sensor	[223]
Feeding force, cutting forces	Dynamometer	[224]
Feeding rate, cutting speed, cutting depth	Force sensor	[225]
Feeding rate, Cutting depths along the radius and axis	Temperature sensor	[226]
Feeding rate, axial and radial depth	Dynamometer	[227]
Speed, cutting depth	Force sensor	[228]
Cutting forces, feeding rate	Temperature sensor	[229]

### 2.3. Advantages and Disadvantages of FPGA-Based Sensors over Traditional Sensors

Due to their unique architecture and capabilities, FPGAs have gained attention in the design and execution of advanced sensing systems. In comparison to traditional sensing devices, which rely on fixed-function hardware, FPGA-based sensors have pros and cons, as compiled in Table 9.

## 3. Enhancing Digital Twin Performance Through FPGA-Based Sensors

### 3.1. Digital Twin Illustration

**Figure 8:**
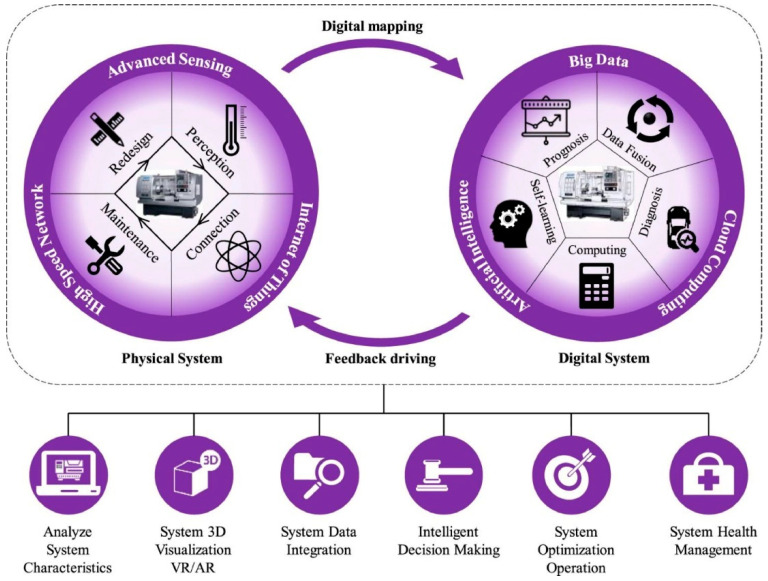
Digital twin for manufacturing [230,237].

A digital twin (DT) is a virtual representation of a physical system, enabling real-time monitoring, evaluation, and optimization [230]. In the case of smart manufacturing (SM), a DT is usually applied to mimic and adjust a manufacturing process, forecast for machine maintenance, and elevate manufacturing efficacy [231]. A DT can also provide a realistic simulation of a given system. DTs usually incorporate advanced tools such as machine learning (ML), data fusion and data analytics, and physics-based models [232,233]. A DT is a vital element for future technologies that can influence various industries. Using the data from physical devices, DT can offer real-time responses, supervise the system performance, and identify possible issues [234]. A DT can also optimize a given system’s operation by suggesting areas for improvement [235]. DT technology has been widely applied to various fields, including automotive, sustainable energy sources, and aerospace [236]. As a result, DT technology may assist in businesses and industrial operations, thus making better decisions and lowering costs. Figure 8 shows the design of a DT for smart manufacturing.

A DT’s essential components are the physical asset or system, a network of sensing devices for continuous improvement of DT and IoT devices that collect data [238]. This real-time data is merged using an effective data collection and amalgamation layer, collecting inputs from various sources, including sensing devices, ERP systems, and various other industrial data repositories [239]. The DT’s heart is the simulation engine, dynamically showing the physical system and allowing predictive evaluation, testing, and performance enrichment. Advanced data analytics, ML algorithms, and feedback loops are also essential to refine the DT model and provide insights for rapid decision-making, resulting in augmented efficacy, lesser interruption, and advanced equipment effectiveness [240,241].

### 3.2. Role of Sensing Devices in a Digital Twin’s Continuous Improvement

Sensing devices (SDs) are critical to the formation and operation of DTs, particularly in smart manufacturing, acting as the key link between the physical and digital assets [242,243]. These devices collect a constant stream of data, indicating the real-time state of a physical asset. This periodic data allows the DT to keep a synced, real-time mirror of the physical system. SDs play a crucial part in the DTs high-fidelity simulation analysis and prediction capabilities.

SDs, by providing operational measurements of important operational characteristics, allow the DT to identify any variations in system performance and predict potential breakdowns [217], enhancing the efficacy of the manufacturing process. The integration of sensing networks and IoT devices broadens the functionality of DTs, allowing for extensive monitoring and supervision of complicated manufacturing processes [244]. Besides, the DTs have been widely explored in various other fields, including vehicle route mapping and control [245]. The data produced by SDs is usually applied in ML algorithms within the DT, allowing for dynamic learning and continual development of the manufacturing process [246]. The efficacy of a DT is inextricably related to the quality and sophistication of the SDs used, since they are important facilitators of real-time, data-driven insights.

### 3.3. Importance of FPGA-Based Sensing Devices in Enhancing Digital Twin Performance

FPGAs can appear as a transformational technology in DT creation, notably in smart manufacturing. Traditional sensing systems are limited by fixed architecture and defined operations. On the other hand, FPGA-based SDs can result in unprecedented flexibility, real-time data processing capabilities, and versatility. These characteristics present FPGAs as ideal candidates for the complicated needs of DTs.

FPGAs can result in significant benefits in instantaneous data processing and synchronization. These devices can be devised for advanced computational jobs immediately at the sensor level, permitting the processing of massive data sets as they are collected. This real-time processing reduces the latency and allows instantaneous updates to the DT, ensuring that the virtual asset properly denotes the physical asset’s real state. This can be a remarkable advantage in high-speed manufacturing environments, where minor delays in data processing can result in major discrepancies between digital and physical assets, risking a DTs reliability. A fundamental advantage of FPGA-based SDs is their flexibility to be reconfigured after implementation. Traditional SDs usually have predefined capabilities and cannot be adjusted for the variable operational conditions. In contrast, FPGAs may be re-programmed, accepting amendments to the sensing system as well as data processing without needing hardware upgrades. This flexibility is critical for sustaining the DTs significance and precision over time since it allows for ongoing adjustment to changing manufacturing processes and the incorporation of emerging sensing modes. Furthermore, FPGAs enable the creation of specialized and tailored designs appropriate to the specific requirements of the DT that is associated with a specific physical asset. This feature enables performance enhancement, such as responsiveness, resolution, and data processing speed, immediately affecting DTs reliability. FPGAs are also able to merge various sensing data streams on a single unit, reducing the need for multiple SDs. This in turn reduces the complexity of the system as well as the cost.

FPGA-based SDs are more resilient than conventional SDs. FPGAs are well-known for their capability to perform in challenging environments, making them ideal candidates for harsh conditions. This robustness allows for the DTs associated with them to preserve consistent dataflow under adverse circumstances, maintaining the dependability of DT. Furthermore, FPGAs parallel data processing abilities permit the concurrent monitoring of many parameters, developing a thorough insight into a manufacturing process, which is critical to the DTs capacity to forecast complex system behaviors. Hence, based on the benefits of FPGAs mentioned above, the incorporation of FPGA-based SDs into DT will elevate their performance through real-time data processing abilities, dynamic reconfigurability, tailored optimization, and robust processing in industrial environments. These benefits present FPGAs a notable selection over traditional SDs, presenting the responsiveness, precision, and trustworthiness required to leverage the DTs in a smart manufacturing environment.

### 3.4. Integration of FPGA-Based Sensors in Enhancing Digital Twin Performance

The incorporation of FPGA-based SDs into DT is a promising measure for advancing system operation, particularly in high-precision and real-time monitoring applications. This section provides case studies demonstrating how FPGA-based SDs can be effectively integrated into DTs to surge their functionality in industrial applications.

#### 3.4.1. Integration of FPGA into Traditional Manufacturing Processes: Milling and Grinding

The system described in utilities a reconfigurable framework using FPGA-based hardware to supervise multiple metal cutting operations. By leveraging a SoC architecture, the system combines three digital signal processors for synchronization, interface, and controlling elements. The VHSIC hardware description language (VHDL) design approach facilitates fluent reconfiguration, yielding an accessible and adjustable system for diverse machining techniques such as milling and grinding. In contrast to typical monitoring techniques, the proposed system can manage signals from various machines with insignificant effort. By targeting average current values compared to complicated measures such as RMS, the system provides quicker monitoring while involving less hardware. Logic comparators facilitate the decision-making procedure by assessing signal limits to verify standard and unsafe settings, permitting the identification of tool wear and excess load situations.

#### 3.4.2. Integration of FPGA into Additive Manufacturing Processes

AM has appeared as a novel strategy in modern manufacturing, yielding values such as rapid prototyping, easy design amendments, and the facility to generate sophisticated internal textures that are difficult to attain with traditional techniques. Regardless of their benefits, AM processes commonly confront internal flaws, compelling real-time monitoring and control to reduce costs by correcting defects promptly. To address this, a CNN trained on the NEU-DET defect dataset is utilized for fault detection, while FPGAs present the computational capability essential for real-time execution. The system realizes 97.90% accuracy in identifying defects by utilizing a binarized neural network appropriate for FPGA operations. This FPGA-based system is extremely flexible and scalable, presenting brilliant performance in defect recognition. Similarly, in laser AM, particularly directed energy deposition, closed-loop controllers are vital for granting steady results across various geometries. The FPGA-based controller improves the laser cladding process by surpassing previous controlling techniques, demonstrating outstanding effectiveness in controlling complicated geometries.

## 4. Leveraging FPGA-Based Sensing Devices for Sustainable D^2^F: A Prospective Approach

### 4.1. D^2^F Framework

A D^2^F modernizes the current manufacturing landscape by connecting different geographically dispersed manufacturing processes and factory environments [247]. This distribution utilizes a networked infrastructure to produce a combined and efficient manufacturing ecosystem. A D^2^F is based on IoT-based devices, cloud computing, AI/ML, and process automation, yielding a flexible and scalable infrastructure. Here, IoT devices act as the D^2^Fs monitoring units, continuously monitoring machine working performance, environmental conditions, and part quality. These devices can handle massive data volumes in real-time [248]. The data collected by IoT devices is vital for retaining working efficiency, identifying defects at early stages, and confirming that each manufacturing stage matches the desired objectives. In this scenario, cloud computing handles the required computational capacity, and storage needs to tackle the massive datasets produced by IoT devices. This also facilitates data consolidation, where all the data are processed and analyzed through a single administrative entity. This consolidation method not only guarantees data stability through the network but also aids in rapid and accurate data interpretation, necessary to make decisions and optimize.

**Figure 9:**
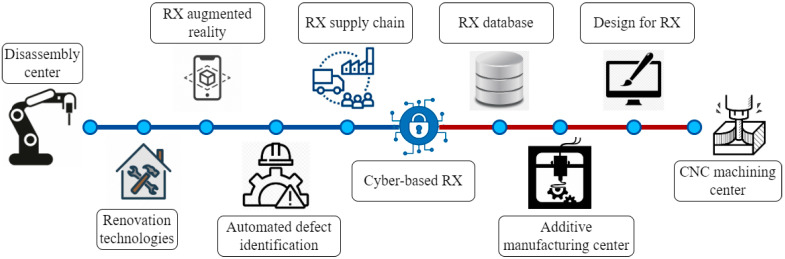
D^2^F framework [247].

AI/ML provides a smart layer to a D^2^F environment. It evaluates accumulated data to detect patterns, foresee tendencies, and thereby improve processes. For example, AI/ML can anticipate when a machine is going to falter based on past heuristics, allowing for preemptive maintenance. It can also adjust manufacturing plans, ensuring that facilities are effectively used with minimal machine downtime. This intelligent (AI/ML) analysis supports constant advancement of the manufacturing processes, adjusting to unique challenges in today’s ever-evolving and rapidly evolving product needs. These systems can modify machine controller settings, re-route jobs, or introduce maintenance practices using real-time datasets and AI-based analyses. All of these ultimately increase the efficiency of manufacturing processes and the reliability of products produced. It is important to mention here that each geographically distributed site acts as a node within the D^2^F model, which is then connected using a digital communication network. These networks will assist in seamless and real-time data exchange and communication. Figure 9 illustrates the D^2^F model. Furthermore, the D^2^Fs capability to function through multiple places offers a substantial benefit in terms of “scalability and resilience”. The D^2^F model can be expanded by adding additional nodes to the network or reallocating jobs among existing sites as needed. This will improve the resilience of the manufacturing process, as the system can adjust to interruptions at any site by reallocating tasks to other nodes inside the network.

### 4.2. D^2^F Sustainability Using FPGA-Based Sensing Devices

In today’s world, the D^2^F is an innovative approach in manufacturing that integrates novel technologies, including IoT-based devices, cloud computing, AI/ML, and automation to allocate and enhance manufacturing processes spanning several geographic locations. The D^2^F is useful for handling the current industry needs, where adaptability, expandability, and durability are vital. For the sustainability of D^2^F, FPGA-based SDs can provide substantial benefits in terms of power efficacy, flexibility, and real-time data acquisition and processing. This section investigates how FPGAs can impact the sustainability of D^2^F.

#### 4.2.1. Power Efficiency and Management

In the manufacturing ecosystem, one of the crucial features contributing towards sustainability is power efficiency [74]. FPGA-based SDs stand out in this domain owing to their capability to execute intricate calculations by utilizing minimum power. Contrasting with established SDs, FPGAs can be adapted to perform specific tasks with elevated productivity, lowering the resources needed for data administration, which is advantageous for D^2^F environments equipped with abundant IoT devices installed at different locations. These devices should be power-efficient for reducing carbon footprints. FPGA-based SDs can handle data locally, lowering the requirement to transfer huge datasets to central cloud servers. These SDs need less energy by committing data processing at the sensor layer. Furthermore, FPGAs can also adjust the energy usage depending on the working requirements, ensuring that the D^2^F can retain a high level of performance at the expense of very low energy needs. This will, in return, contribute towards the sustainability of the D^2^F.

#### 4.2.2. Instantaneous Monitoring and Dynamic Control

FPGA-based SDs can increase the D^2^F sustainability through their capability to yield instantaneous monitoring and dynamic control. In a dispersed manufacturing ecosystem, the capability to supervise operations instantly is vital for preserving effectiveness and lessening waste. FPGAs, with their inherent parallel processing abilities and low latency data administration, facilitate rapid response and corrections, constantly confirming that processes are operating within their optimized targets at all times [58]. For instance, within D^2^F, FPGA-based SDs can identify abnormalities in manufacturing processes and start counteractive actions without delay. This instantaneous reaction decreases the probability of flaws, material waste, and power misuse, further supporting the sustainability of D^2^F.

#### 4.2.3. Increased Versatility and Lifespan

The D^2^F sustainability is also supported by the versatility and lifespan of FPGA-based SDs. Compared to conventional SDs having fixed functionalities, FPGAs are reconfigurable to adjust to any new variations within a manufacturing process [82]. This flexibility expands the duration of the sensor’s life, lowering the requirement for repeated substitutions, thereby reducing electronic waste. As manufacturing processes advance, FPGAs can be improvised to adjust to unique sensing modules, allowing the D^2^F to respond to the adjusting demands without substantial hardware changes. This flexibility not only supports the working sustainability of the D^2^F but also supports overarching environmental objectives linked with hardware manufacturing and future factory upgrades.

#### 4.2.4. Data-Informed Decisioning and Process Improvement

In today’s manufacturing regime, sustainability is driven by data, with modern data analytics performing a decisive role in process and resource optimization. FPGAs can instantaneously process data, permitting an instant application of AI/ML insights and supporting constant advances in manufacturing efficacy. For D^2^F, this analytics-based improvement can produce substantial decreases in power use, material waste, and manufacturing time and costs. FPGA-based SDs can reduce the environmental effect of manufacturing processes by ensuring that the distributed manufacturing processes perform at peak efficiency with all factory equipment and resources being constantly, consistently, and safely utilized. Additionally, the understandings derived from data processing can be applied to improve manufacturing strategies, catering to long-term sustainability by supporting optimized resource utilization.

#### 4.2.5. Robustness of D^2^F for Sustainable Operations

The robustness presented by FPGA-based SDs is critical for D^2^F sustainability. The capability to scale up operations at various locations while keeping efficiency high with modest environmental effects is a prime benefit of D^2^F. FPGAs equipped with robustness and flexibility can facilitate this scalability by supplementing a broad scale of monitoring and analysis demands across diverse sites. The robustness of FPGA-based SDs, specifically their capability to work in harsh environments, confirms that the D^2^F can preserve eco-friendly operations under adverse conditions. This will reduce the probability of idle time and manufacturing losses related to higher resource usage and ecological impact. Hence, FPGA-based SDs can provide effective operations across a dispersed network and can play a critical role in the sustainability of D^2^F.

#### 4.2.6. Challenges in FPGA-Based Sensors for D^2^F Systems

Regardless of the great capability for FPGA-based SDs to revolutionize the D^2^F manufacturing systems, several barriers must be resolved to ensure their effective integration. Addressing these obstacles is critical for executing the FPGA-based SDs in the D^2^F systems. While the literature demonstrates the characteristics of FPGA technology, a detailed investigation is needed along with the mitigation techniques to fulfill the gap between theoretical advancement and actual implementation. The following sections highlight the challenges in FPGA-based sensors for D^2^F systems.

- Integration and Interoperability

Leveraging the FPGA-based SDs into the dispersed manufacturing settings is difficult owing to the varying system architectures involved. FPGA platforms differ in terms of design, programming languages, and hardware abilities, causing compatibility issues [249]. Moreover, facilitating smooth interoperability across multiple manufacturing systems, which contain both legacy infrastructure and modern manufacturing tools, remains a foremost challenge [250].

- Real-Time Data Processing Across Networks

FPGA SDs are intended for rapid data capture and processing. However, their efficacy in distributed manufacturing environments is restricted owing to the restrictions of real-time operation across large networks [251]. Synchronizing several FPGA-based sensors in a scattered digital factory can also result in processing delays, bandwidth constraints, and data inconsistencies, contributing to lessening the system efficiency. These challenges must be resolved before their implementation in the D^2^F environment.

- Scalability

Extending FPGA-based SDs to meet the requirements of large-scale manufacturing environments proposes substantial obstacles. As distributed networks increase in size and complexity, restraints in FPGA hardware, such as inadequate memory capability and processing characteristics, become more evident [252]. Furthermore, modifying software techniques to support scalable functionality increases complexity.

Cost and Availability Obstacles

Employing FPGA-based SDs in distributed systems requires substantial financial investment, including the initial cost of hardware, continuing operational expenditures, and the requirement for dedicated expertise in design, programming, and maintenance [253]. These obstacles can make it challenging for SMEs to execute such technology.

- Reliability in Manufacturing Environments

For D^2^F, FPGA-based SDs operate constantly in challenging manufacturing environments that include electromagnetic interference, temperature variations, and mechanical vibrations [254,255]. To complete durability in these circumstances, extensive protective methods are required, which might elevate the system’s complexity and expense.

- Security Vulnerabilities

Cybersecurity attacks are a typical problem for distributed manufacturing systems, and FPGA-based SDs are also vulnerable to these attacks [256]. To safeguard these systems, encryption techniques and secure communication protocols must be developed for FPGA-based SDs. Nonetheless, progress in this domain is still restricted in the context of D^2^F, causing potential weaknesses for cyber security.

- Lack of Standardized Frameworks

The lack of standardized protocols for FPGA-based SDs in manufacturing systems impedes their prevalent utilization [257]. Reliable guidelines for communication, data formatting, safety, and performance assessment are lacking, hindering incorporation and compliance efforts.

- Development and Debugging

FPGA development is intrinsically complex since it needs hardware-specific programming and optimization [258]. FPGAs need designers to comprehend low-level hardware particulars, making the development process longer. Furthermore, debugging FPGAs is tedious as it regularly involves hardware and temporal intricacies that are difficult to detect using software tools. Comprehensive validation is also hindered by the limited observability of internal states during the process, necessitating the use of advanced simulation tools and real-time analyzers to ensure system stability and accuracy.

## 5. Conclusions and Future Outlook

This review provides an in-depth analysis on the integration of FPGA-based sensing devices (SDs) within D^2^Fs, highlighting their role in expanding sustainability and improving operations within manufacturing systems. The exclusive features of FPGA-based SDs, including reconfiguration, modest energy utilization, and rapid data processing, establish them as vital tools for instantaneous supervision and control of D^2^Fs. These SDs enable real-time data management and robust reconfiguration, necessary for continuing the accuracy of DTs associated with the proposed D^2^F, confirming that the virtual assets accurately represent their physical counterparts.

FPGA-based SDs not only adjust the efficacy and sustainability of the manufacturing processes but also impact the scalability of D^2^Fs. Their capacity to perform efficiently in tough conditions and adjust to varying and evolving conditions prolongs the lifetime of SDs, lowering the requirement for repeated substitutions and reducing waste. This flexibility, integrated with the elevated accuracy and instantaneous monitoring abilities of FPGA-based SDs, marks them as a crucial technology for innovative intelligent manufacturing.

Prospective research is required to investigate the capacity of FPGA-based SDs in intricate and data-intensive uses within D^2^Fs. As digital manufacturing is evolving continuously, the incorporation of next-generation sensing technologies will be critical in steering sustainable and robust manufacturing ecosystems. The constant amendment in FPGA technology, alongside improvements in AI/ML and IoT tools, will absolutely lead to modern and effective D^2^Fs, able to meet the increasing demands of tomorrow’s industry.

Furthermore, despite the increased interest of FPGAs for SDs, there is still a gap in evaluating their performance compared to traditional SDs. Performance parameters, including response time, data processing speed, and accuracy, are rarely evaluated in a systematic manner, limiting the widespread use of FPGA-based SDs. To address this research gap, future research should incorporate one-on-one comparisons of FPGA-based SDs and traditional SDs across key performance measures. Experiments or simulation analyses with numerous situations and processing conditions are needed to acquire a systematic capacity of their relative strengths and constraints. Such research would not only emphasize the advances of FPGA-based SDs but would also benefit to improve them for certain use conditions.

## Figures and Tables

**Table 2:** Pros and cons of FPGA-based hard and soft processors.

Item	Hard Processor	Soft Processor
Illustration	Embedded physical core processors built into the FPGA chip	Equipped with configurable processing cores developed using FPGA logic blocks
Operation	Elevated working efficiency with optimized speed and power utilization	Less working efficiency due to dependency on FPGAs logic resources
Energy utilization	Less energy consumption, as they have been optimized for a particular task	Increased energy utilization compared to hard processors
Resources	Do not exhaust FPGA logic-based resources, releasing space for other purposes	Consume FPGA logic-based assets, reducing accessibility for the rest of the tasks
Flexibility	Do not provide flexibility as they have been fixed to FPGA fabric	Flexible and customizable based on the requirements
Seamless integration	Uniform integration with FPGA fabric, managing effective operation	Require additional effort related to design for integration into FPGA devices
Engineering difficulty	Easier to utilize with standardized tools and computing system	Necessitates more complex advancement and customization
Cost	Require high cost for fixed nature of processor	Less cost as they are still within FPGA resources
Scalability	Restrained scalability by the FPGA architecture	High scalability as required by FPGA resources
Usage suitability	Ideal for high performance as well as effective utilization	Ideal for applications requiring iterative design and testing

**Table 9:** A comparative analysis of FPGA and traditional sensing devices.

FPGA-Based Sensing Devices	Traditional Sensing Devices
Highly customized in real-time for unique applications	Fixed function and need hardware reform
Provides parallel processing to tackle large datasets	Limited processing due to the sequential nature of data handling
Provide deterministic working performance	Variable working performance
Less latency and high-throughput, and can provide analysis in real-time	Less beneficial for real-time processing due to reduced throughput and increased latency
Energy efficient in performing tasks when optimized	Less power consumption with varying efficacy
Integrate multiple operations in a single unit	Require multiple components
Requires HDLs and digital logic design expertise	Utilize simple controllers
Elevated higher costs for design and development	Less cost when used on small scale
If not utilized properly, it may result in higher energy consumption	If utilized on a smaller scale, it may result in less energy consumption
Provides challenges in achieving analog signal precision	Excels in providing analog signal processing precision
Results in interoperability issues due to non-standardization	Integrates with ease into systems due to standardization

## Data Availability

Not applicable.
